# A Comparative Identification of Ochratoxin A in Longan Fruit Pulp by High Performance Liquid Chromatography-Fluorescence Detection and Electron Spray Ionization-Mass Spectrometry

**DOI:** 10.3390/molecules15020680

**Published:** 2010-01-29

**Authors:** Jing Li, Haihui Xie, Bao Yang, Xinhong Dong, Linyan Feng, Feng Chen, Yueming Jiang

**Affiliations:** 1 South China Botanical Garden, Chinese Academy of Sciences, Guangzhou 510650, China; E-Mails: ruochenjl@163.com (J.L.); xiehaih@scib.ac.cn (H.X.); yangbao@scib.ac.cn (B.Y.); donganny@glite.edu.cn (X.D.); fenglinyannihao@163.com (L.F.); 2 Department of Food Science and Human Nutrition, Clemson University, Clemson, SC 29634, USA; E-Mail: fchen@clemson.edu (F.C.)

**Keywords:** ochratoxin A, longan, fruit, pulp, solid-phase extraction, HPLC-FD, ESI-MS

## Abstract

Harvested longan (*Dimocarpus longan* Lour.) fruit are susceptible to decay caused by both bacterial and fungal infections. Ochratoxin A (OTA) is a kind of mycotoxin produced by a number of fungi. In this study, OTA was extracted from longan fruit pulp by 80% methanol and then loaded on C-18 solid-phase extraction columns. The extract solution was then analyzed by high-performance liquid chromatography - fluorescence detection (HPLC-FD) and an electron spray ionization-mass spectrometry (ESI-MS), respectively. The HPLC-FD analysis showed that a compound similar to OTA might exist in longan fruit pulp, but further analysis by the ESI-MS method demonstrated that OTA was not present in the longan pulp, indicating that the presence of OTA in longan fruit pulp detected by the HPLC-FD analysis needed to be confirmed by the ESI-MS method.

## 1. Introduction

Mycotoxins, the secondary metabolites of fungi, that include a large heterogeneous group of substances such as aflatoxins, ochratoxin A (OTA), patulin, and other toxins [1], can cause deleterious effects on animals and, in some circumstances, on human beings [2]. As a result, mycotoxin contamination in foods causes considerable economic losses. Particularly, aflatoxins in dried fruit and *Fusarium* sp. related toxins in cereal (particularly maize, wheat, and barley), and OTA in cereal and coffee are among the toxic species of the greatest economic and safety concern [3]. Consequently, many efforts have been made to establish relevant analytical methods and regulatory limits for mycotoxins. Currently, over 100 countries have regulations regarding mycotoxins in feed, in which 13 mycotoxins or mycotoxin groups are covered [4].

Ochratoxin A, produced by a number of fungal species such as *Penicillium* sp. and *Aspergillus* sp., exhibits nephrotoxic, teratogenic, immunotoxic, cancerogenic and possibly neurotoxic properties [5,6,7]. This toxic chemical can contaminate many foods including cereal grains, vegetables, beverages, fresh fruits and dried fruits as well as animal feed [1,2,8,9]. Engelhardt *et al*. [10] analyzed different fruits such as peaches, cherries, strawberries and apples after the removal of rotten tissues and found only the presence of OTA at levels up to 2.71 mg/kg in cherry and 1.44 mg/kg in tomato. Peaches and apples were also subject to ochratoxin A contamination but to a lesser degree [10]. These results indicated that damaged or pathogen-infected fruits could be easily contaminated with ochratoxin A. Thus, investigations into the presence of OTA in harvested fresh fruit caused by latent infection of pathogens are needed. 

Several reviews of the analytical methods for determining OTA in foods and feeds are available [11,12,13,14]. These methods are largely based on high-performance liquid chromatography with fluorescence detection (HPLC-FD) after extensive sample clean-up using methods such as liquid-liquid extraction, solid-phase extraction or antibody-based immunoaffinity chromatography [9,11,12,13,14]. However, analytical problems such as co-elution of interfering compounds and retention time shifts can lead to erroneous positive or negative results. These problems could be overcome by the direct coupling of liquid chromatography (LC) with mass spectrometry (MS) using electron spray ionization (ESI) [9] due to its powerful sensitivity, accuracy and capability of chemical identification via a compound’s mass spectrum. To the best of our knowledge, the use of mass spectrometric detection for the analysis of OTA has only been described for a few samples such as human blood, beer, coffee and wine in a few publications [15,16].

Longan (*Dimocarpus longan* Lour.) is a nutritional subtropical fruit that is preferably eaten as a fresh product [17]. This fruit plant is widely distributed in some Asian countries such as China, Vietnam and Thailand [18]. However, longan fruit is very perishable because of both bacterial and fungal infections, resulting in serious quality deterioration [18]. The pathogens include *Penicillium* sp., *Rhizopus* sp., *Asperillus* sp., *Alternaria* sp., *Lasiodiplodia theobromae*, *Pestalotiopsis* sp., *Cladosporium* sp., *Fusarium* sp., *Colletotrrichum gloeosporioides*, *Geotrichum candidum*, *etc*. [19,20,21]. Longan fruit can also suffer from latent infection by pathogens before harvest [18], which may result in mycotoxin contamination of the fruit after harvest. Therefore, it is important to establish a rapid, accurate and feasible analytical method for evaluation of OTA in logan fruit in an effort to reduce economic losses. The objective of this study was to investigate the contamination of OTA in longan pulp by the HPLC-FD or ESI–MS method, and compare the two methods.

## 2. Results and Discussion

A properly optimized method is necessary to ensure good accuracy and precision, together with reasonable detection limits. Although the procedures used to isolate and purify the OTA can differ substantially, the protocols to detect OTA in various fruits generally consist of extraction, purification, and finally OTA identification [15]. Usually, solid samples are extracted using acetonitrile-water, methanol-water or even chloroform to enhance the solubility and extraction efficiency of OTA [22]. In this study, methanol-water and dichloromethane were used as the extraction solvents. In addition, the complexity of the fruit sample requires a pre-treatment step to enable good isolation of OTA. Considering the expensive cost of using of immunoaffinity columns, solid-phase extraction was chosen for purifying OTA from the dried longan pulp in this study because this treatment was found to have a similar recovery compared to the immunoaffinity method. Previous analytical methods for the identification of OTA in fruit pulps and juices often used reversed-phased high-performance liquid chromatography coupled to fluorescence detection [12,16], but this method could display some analytical problems such as co-elution of interfering compounds or retention time shifts that could lead to erroneous positive or negative results [9]. In recent years, detection of OTA in beer and wine by LC/MS has been developed by Bacaloni *et al*. [23] and Reinsch *et al*. [24]. 

**Figure 1:**
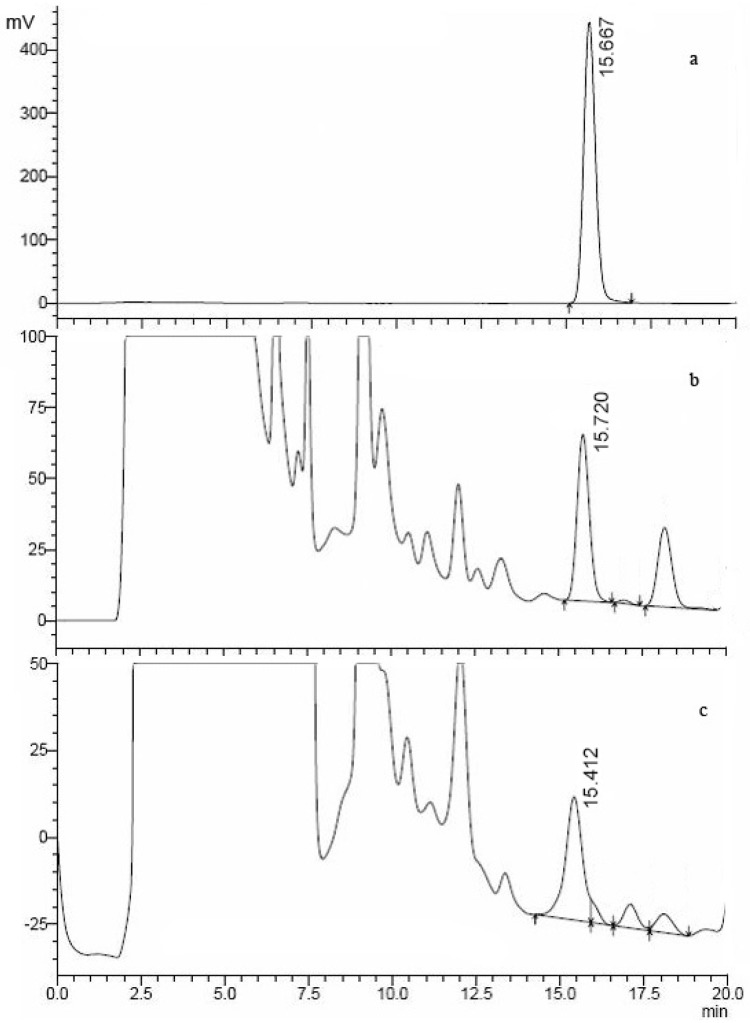
HPLC-FD (λex = 333 nm and λem = 460 nm) of ochratoxin A standard (a) and the pulp extracts from good (b) and diseased (c) longan fruits.

**Figure 2:**
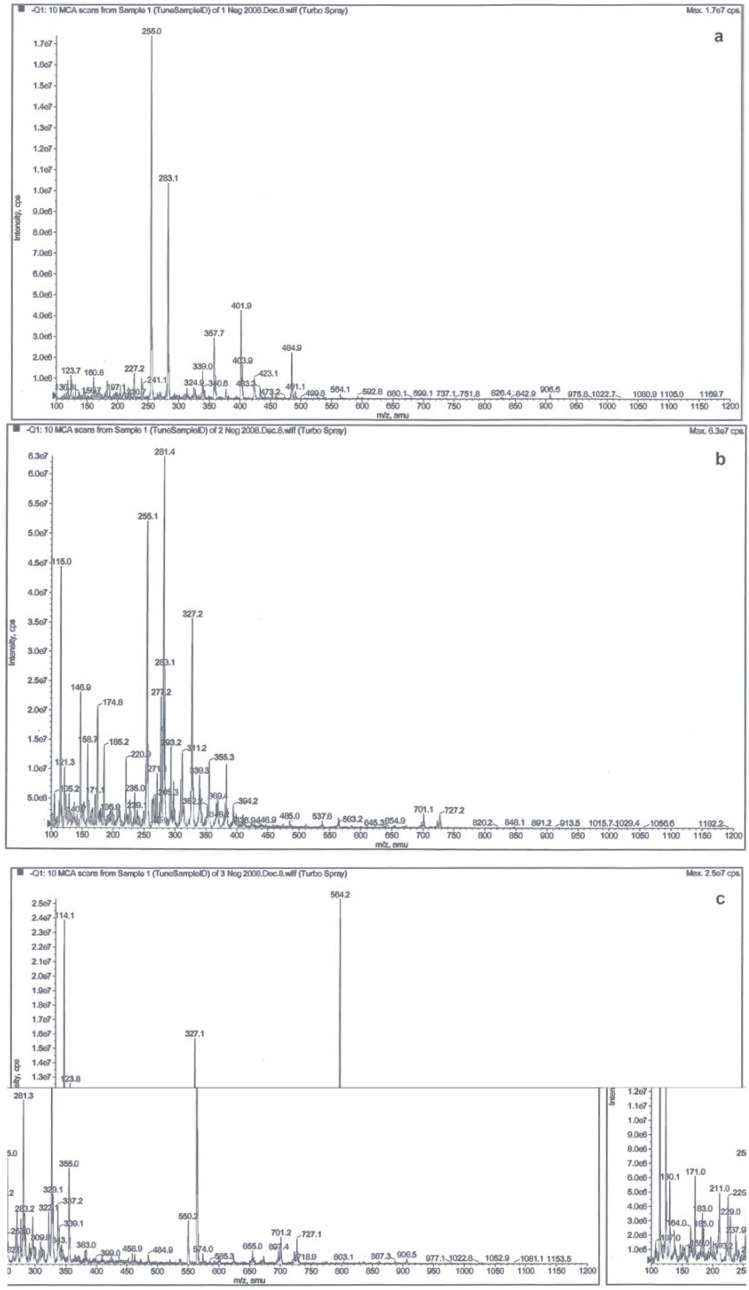
ESI mass spectra in the negative ion mode [M-H]^-^ of 1 μg/mL standard ochratoxin A (a) and the pulp extracts from good (b) and diseased (c) longan fruit.

Figure 1 shows chromatograms of an OTA standard and two longan pulp samples. The HPLC-FD analysis showed that the standard OTA had a retention time at 15.667 min (Figure 1as were observed at 15.72 (Figure 1b) and 15.412 min in the longan pulp from good fruit and diseased fruit (Figure 1c analogues of OTA might be present in good and diseased pulp. However, the retention time of OTA could vary under the different conditions and instruments used, and the related factors include the eluent, solvent, instrumental error or error in operation [9,15]. Timperio *et al*. [15] investigated the effect of pH on the spectroscopic emission of OTA during elution and found that the elution time of OTA increased from 4 to 8 min when the eluent was used from acid pH to alkaline solution, with an enhanced OTA fluorescence. In this study, two compounds similar to the retention time of ochratoxin A standard were observed in the good fruit pulp and diseased fruit pulp but they exhibited different [M-H]^- ^peaks, as indicated in Figure 2. Thus, the identification of the two compounds was required. 

The negative ESI-MS of the standard OTA exhibited the [M-H]^- ^peak at m/z 402 and [M-CO_2_-H]^- ^peak at m/z 358 (Figure 2a), which was in agreement with the results reported by Bacaloni *et al*. [23] and Vatinno *et al*. [16]. However, no peaks were observed in the negative ESI-MS, suggesting that OTA were not present in longan pulp samples or the level of OTA present in the pulp could be too low to be detected. Furthermore, the negative ESI-MS of the longan pulp from good fruit exhibited a [M-H]^- ^peak at m/z 327 and a [2M-H]^- ^peak at m/z 655 with a molecular weight of 328 (Figure 2b), but the pulp sample from the diseased fruit showed an abundant [M-H]^- ^peak at m/z 564 with a molecular weight of 565 (Figure 2c). Thus, differences in these major [M-H]^- ^peaks with regards to molecular weights existed between good longan fruit and diseased longan fruit, suggesting the presence of different compounds. 

Many pathogens can infect longan fruit before and after harvest. About 106 species of microorganism have been isolated from longan fruit, comprising of 36 bacteria, 63 molds and seven yeast species [19]. Fungal pathogens such as *Penicillium* sp., *Rhizopus* sp., *Asperillus* sp., *Alternaria* sp., *Lasiodiplodia theobromae*, *Pestalotiopsis* sp., *Cladosporium* sp., *Fusarium* sp., *Colletotrrichum gloeosporioides* and *Geotrichum candidum* were all identified in rotten longan fruit tissues [19,20,21]. Many mycotoxins can be produced by *Penicillium* sp., *Asperillus* sp., *Alternaria* sp. and *Fusarium* sp. [1,2] while a toxin compound produced by *Lasiodiplodia theobromae* was identified as (3S,4R)-3-carboxy-2-methylene-heptan-4-olide [25]. In this study, although no OTA was detected in longan pulp, it is necessary to further identify other toxic compounds possible present due to latent pathogen infection. In addition, this technique can also help to determine the presence of OTA in other fruit samples.

## 3. Experimental

### 3.1. Plant materials

Fresh mature longan fruit (*Dimocarpus longan* Lour. cv. Shixia) were obtained from a local commercial orchard near Guangzhou (China). Fruits without visual defects were selected for uniformity of weight and shape and recorded as the good ones. Fruits with obvious mold growth were selected as the diseased ones. These fruits were peeled, and then pulp was separated from seeds, collected and dried for 10 h by using a constant temperature cabinet set at 70 °C. The dried pulp was stored in a refrigerator at -20 °C prior to analysis.

### 3.2. Chemicals and reagents

Supelclean^TM^ C-18 solid-phase extraction tubes were obtained from Supelco (Supelco, Sigma-Aldrich, USA). Standard OTA was purchased from Sigma–Aldrich Co. (Canada). Standard 1 and 5 μg/mL OTA solutions were prepared using HPLC grade methanol (Sigma, USA). Water used in this study was purified with a Milli-Q system (Millipore, Bedford, MA, USA). All reagents used in this study were the analytical reagent or HPLC grade. 

### 3.3. Extraction of OTA from the dried longan pulp

The extraction procedure was carried out according to the methods described by Sáez *et al*. [9] and Timperio *et al*. [15], with some modifications. The dried longan pulp (40 g) was extracted with methanol-water (80:20, v/v, 100 mL) by blending for 3 min at 10,000 rpm. The extract was filtered through a filter paper (Whatman glass microfiber GF/A), and then the residue was washed once with methanol/water (80:20, v/v, 100 mL) and filtered. The filtrates were combined and then concentrated to a small volume on a rotary evaporator (BC-R203, Shanghai Biochemical Equipment Co., Shanghai, China) at 50 °C under vacuum. The concentrated filter was dissolved in Milli-Q water (100 mL) and then half of the solution was extracted three times with dichloromethane (25 mL). The dichloromethane layers were combined and evaporated to dryness under a gentle N_2_ stream at 30 °C. The residue was dissolved in methanol (5 mL) and then the extract solution was stored in a refrigerator at -20 °C until used. 

### 3.4. Purification of OTA extraction using C-18 solid-phase extraction column

According to the method described by Hernández *et al*. [12], the C-18 solid-phase extraction column was treated with methanol (5 mL) and then equilibrated with Milli-Q water (5 mL). Longan extract solution (2 mL) was loaded onto the column and washed sequentially with Milli-Q water (2 mL) and methanol/water (60/40, v/v, 2 mL), then the elute was discarded. After air-drying the column, elution of OTA was carried out with acetonitrile (2 mL) and the eluate was filtered through 0.45 μm a Millipore filter membrane before injection into the HPLC system. All the samples were analyzed in duplicate.

### 3.5. HPLC-FD analysis

According to the method of Visconti *et al*. [26], OTA was analyzed on a reversed-phase HPLC system (Shimadzu LC-20A, Shimadzu, Japan) using a Diamonsil C-18 column (250 × 4.6 mm i.d. and 5 μm particle size) and a RF-10 AXL fluorescence detector. The excitation and emission wavelengths were 333 and 460 nm, respectively. The mobile phase was acetonitrile/water/acetic acid (49.5:49.5:1, v/v/v). The injection volumes of sample and standard OTA solution (5 μg/mL) were 100 and 25 μL, respectively. Samples were eluted at a flow rate of 1.0 mL/min. Under these conditions, the OTA analysis was conducted within a time of less than 20 min. 

### 3.6. ESI–MS identification

The ESI-MS analysis conditions were based on the method described by Reinsch *et al*. [24] and Timperio *et al*. [15]. Mass spectrometry was performed on a SCIEX API 2000 LC/MS/MS System (ABI, USA) equipped with an electron spray ionization (ESI) source operated in negative mode. Samples were directly injected into the instrument after being filtered through a 0.45 μm Millipore filter membrane. The inlet flow was adjusted to 5 μL/min and nitrogen was used as curtain gas whereas air was used as the ion source gas. Declustering potential was set at -45 V. The samples were analyzed by using a turbo ionspray ionization source at a voltage of -4.5 kV on the ESI interface. Continuous mass spectra were recorded by the accumulation of 10 MCA (multiple channel acquisition) scanning over mass ranges of m/z 100–1,200.

## 4. Conclusions

The current HPLC-FD method is quick but not the most accurate test for dried fruits. Sometimes a sensitive confirmation technique such as LC/MS/MS is necessary to assure peak purity at the low level. The study indicated that the presence of OTA in longan fruit pulp detected by the HPLC-FD analysis needed to be further confirmed by the ESI-MS method, which gave negative results. The work can also help to detect OTA in other fruits.
